# Regular nicotine intake increased tooth movement velocity, osteoclastogenesis and orthodontically induced dental root resorptions in a rat model

**DOI:** 10.1038/ijos.2017.34

**Published:** 2017-09-29

**Authors:** Christian Kirschneck, Michael Maurer, Michael Wolf, Claudia Reicheneder, Peter Proff

**Affiliations:** 1 Department of Orthodontics, University Medical Centre of Regensburg, Regensburg, Germany; 2 Department of Oral and Maxillofacial Surgery, University Medical Centre of Regensburg, Regensburg, Germany; 3 Department of Orthodontics, Rheinische Friedrich Wilhelm University of Bonn, Bonn, Germany

**Keywords:** dental research, inbred Fischer344, nicotine, orthodontics, rats; root resorption, tooth movement

## Abstract

Orthodontic forces have been reported to significantly increase nicotine-induced periodontal bone loss. At present, however, it is unknown, which further (side) effects can be expected during orthodontic treatment at a nicotine exposure corresponding to that of an average European smoker. 63 male Fischer344 rats were randomized in three consecutive experiments of 21 animals each (A/B/C) to 3 experimental groups (7 rats, 1/2/3): (A) cone-beam-computed tomography (CBCT); (B) histology/serology; (C) reverse-transcription quantitative real-time polymerase chain reaction (RT-qPCR)/cotinine serology—(1) control; (2) orthodontic tooth movement (OTM) of the first and second upper left molar (NiTi closed coil spring, 0.25 N); (3) OTM with 1.89 mg·kg^−1^ per day s.c. of L(−)-nicotine. After 14 days of OTM, serum cotinine and IL-6 concentration as well as orthodontically induced inflammatory root resorption (OIIRR), osteoclast activity (histology), orthodontic tooth movement velocity (CBCT, within 14 and 28 days of OTM) and relative gene expression of known inflammatory and osteoclast markers were quantified in the dental-periodontal tissue (RT–qPCR). Animals exposed to nicotine showed significantly heightened serum cotinine and IL-6 levels corresponding to those of regular European smokers. Both the extent of root resorption, osteoclast activity, orthodontic tooth movement and gene expression of inflammatory and osteoclast markers were significantly increased compared to controls with and without OTM under the influence of nicotine. We conclude that apart from increased periodontal bone loss, a progression of dental root resorption and accelerated orthodontic tooth movement are to be anticipated during orthodontic therapy, if nicotine consumption is present. Thus patients should be informed about these risks and the necessity of nicotine abstinence during treatment.

## Introduction

Deleterious effects of nicotine and tobacco smoke are often investigated and discussed regarding their interrelationship with cancer as well as chronic conditions of the cardiovascular system such as atherosclerosis and respiratory diseases such as chronic obstructive pulmonary disease (COPD), which can affect adults and children all the same.^1^ However, limited attention has yet been given regarding possible effects on the oral or stomatognathic system, in particular in association with orthodontic treatment.

Teeth are linked to their surrounding alveolar bone socket of the jaw via connective tissue, the periodontal ligament, also known as dento-alveolar joint or gomphosis.^2^ In the dental specialty of orthodontics, tooth movement—therapeutically induced by fixed or removable orthodontic intraoral appliances—is performed to enhance position and alignment of permanent teeth for improved masticatory and phonetic function, psychological and esthetic reasons. To this end, a physiological, defined mechanical force is applied to the respective teeth, which results is the creation of tension and pressure zones within the periodontal ligament.^3^ This triggers a pseudo-inflammatory, immunological, multicellular process instigated by periodontal fibroblasts,^4^ resulting in increased osteoclast differentiation and bone resorption in direction of movement as well as bone formation by osteoblasts in zones of tension.^3^ In some cases, osteoclast activity during orthodontic tooth movement also turns against the tooth itself, causing orthodontically induced inflammatory dental root resorptions (OIIRR), which are a rather frequent and unpredictable side effect during orthodontic treatment of varying severity and unknown etiology.^5, 6^

**Figure 1:**
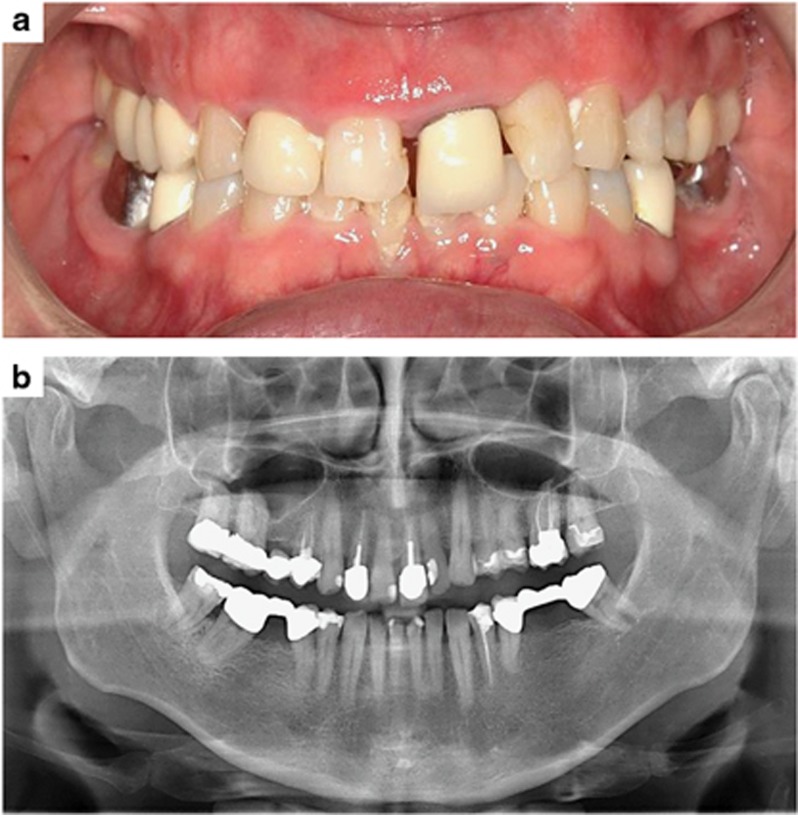
**Adult orthodontic patient with a long-term consumption of more than 10 cigarettes per day, requiring an orthodontic correction of the dental deep bite and misaligned first upper incisor**. (**a**) Intraoral view; (**b**) panoramic radiograph of the dentition and alveolar jaw bone. The gingival tissue shows signs of inflammation and a general horizontal periodontal bone loss due to a chronic nicotine-induced periodontitis is evident.

According to the World Health Organization, the use of tobacco is “the single most preventable cause of death and disease” worldwide and “Europe has the highest prevalence of tobacco smoking among adults (28%) and some of the highest prevalence of tobacco use by adolescents” (11%–12%) with prevalence rates in America ranging between 13% and 22% (adults).^7^ With adolescents constituting the majority of orthodontic patients and the number of adults requiring orthodontic treatment continuously rising,^8^ orthodontists are frequently confronted with patients smoking regularly^9^ (Figure 1).

Of the more than 4000 chemicals contained within tobacco smoke, nicotine seems to play a major role regarding tobacco-induced pathobiological effects on the periodontal apparatus.^10, 11, 12, 13, 14^ Nicotine has already been shown to cause an inflammation of the periodontal ligament (periodontitis) and subsequent loss of alveolar jaw bone with increased risk of tooth loosening.^12, 15^ This can increase the malposition and misalignment of permanent teeth by pathological tooth migration,^16^ which at the same time also increases the need for orthodontic treatment. Recently it has been shown by Akinkugbe *et al.*^17^ that even environmental tobacco smoke (ETS) exposure is associated with periodontitis. As a result, even nonsmokers may be at risk to incur nicotine-associated deleterious effects regarding their teeth, the periodontal apparatus and thus orthodontic treatment.

Although for safety reasons orthodontic patients are generally advised to refrain from smoking during therapy, it is widely unknown at present, whether and which undesired side effects can be expected during orthodontic treatment, if the patient should choose to continue or resume smoking or is exposed to nicotine during therapy either by environmental tobacco smoke or a nicotine patch during smoking cessation therapy. This happens in many cases and mostly unbeknown to the orthodontist. In previous studies, we could already show that orthodontic tooth movement during acute periodontitis or nicotine exposure increased associated periodontal bone loss respectively,^10, 18^ whereas further effects in the context of orthodontic treatment and underlying molecular and cellular mechanisms remain largely unknown.

In our study we thus investigated in a rat model, if chronic nicotine exposure at a dosage corresponding to that of an average European smoker affects the velocity of orthodontic tooth movement, associated undesired OIIRR as well as inflammation and osteoclast activity in the periodontal ligament.

## Materials and methods

### Experimental animals and housing

As experimental animal strain male inbred Fischer344 (F344) rats (*Rattus norvegicus BERKENHOUT*) were chosen, purchased from the Charles River Laboratories (F344/DuCrl, Sulzfeld, Germany) at an age of 6 weeks at shipment and mean gross body weight of (260±15) g at the inception of orthodontic tooth movement. The strain has been used in studies on nicotine before^10, 19^ and was selected for its genetic consistency to minimize biological variability.^2, 18^

The animals were housed in a conventional animal laboratory. They were exposed to a constant noise-free environment (55%±10% humidity, (21±1) °C room temperature, 25 Pa overpressure, 16 air changes per hour) and a day-night rhythm of 12:12 h with the light phase ranging from 7 am to 7 pm. 4–5 animals of differing experimental groups (one per group) shared one type IV metal grid polycarbonate cage (Makrolon) to minimize possible bias. Bedding consisted of fiber soft wood shavings (germ reduced) type ¾, which were changed once a week (Altromin, ssniff Spezialdiäten GmbH, Soest, Germany). Tap water, changed twice a week, and a standard rat maintenance diet (V1535, ssniff, Soest, Germany) were both administered *ad libitum*. Beginning with experimental tooth movement, the dry food pellets were mixed to mash daily. This was done to minimize the chance of mechanical damage to the orthodontic appliance as well as trauma to the periodontal apparatus leading to a loosening of teeth with reduced attachment.^10, 20^ A health monitoring according to Federation of European Laboratory Animal Science Associations(FELASA) guidelines was maintained (stock sentinels, serological and microbiological testing) as well as a suitable acclimatization period after shipment to minimize stress-induced experimental bias.

### Experimental design and procedures

#### Design, Sample size and Allocation

63 F344 rats in total were randomly allocated to three experimental groups (1–3; *n*=7) within three successive experiments of 21 rats (A/B/C) each (block randomization, concealed random allocation sequence):

1. Control group (untreated).
2. Orthodontic tooth movement (OTM) of the left upper first/second rat molars.
3. OTM with a subcutaneous nicotine administration of 1.89 mg per day per kg gross body weight.

Experiment A consisted of cone-beam-computed tomography (CBCT) radiological imaging at baseline and after 14 as well as 28 days of OTM to quantify orthodontic movement velocity. Experiment B was performed to assess OIIRR and osteoclast activity within the periodontium (histomorphometry, tartrate-resistant acid phosphatase(TRAP) histochemistry) after 14 days of OTM as well as systemic effects of nicotine by means of a serological interleukin(IL)-6 analysis. Experiment C was performed to conduct a gene expression analysis of osteoclast (CTSK/CLCN7) and inflammatory (IL-1β/-6/-8) markers within the dental-periodontal tissue after 14 days of OTM by means of reverse-transcription quantitative real-time polymerase chain reaction(RT–qPCR). In addition, serum cotinine concentration was quantified to evaluate nicotine bioavailability.

The time periods and sample size of experimental groups selected were established before as suitable for pharmacological-toxicological orthodontic studies on rats.^2, 10, 21^ Each intervention was performed by one researcher at approximately the same time for animals of the same cage. Groups 1 and 2 (control/OTM) were also utilized as control groups in other concurrently performed animal studies, reducing the total number of animals required (3R).

#### Nicotine administration and monitoring

**Figure 2:**
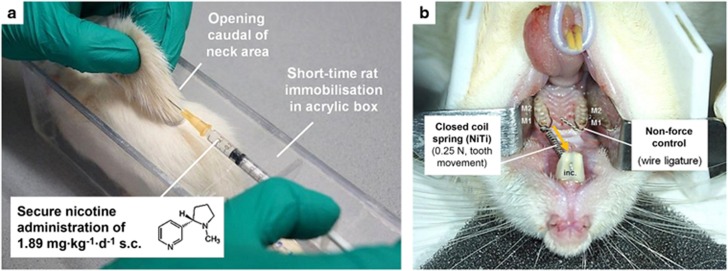
**Subcutaneous administration of L(−)-nicotine at a dosage of 1.89 mg per kg gross body weight per day (a) and experimental orthodontic tooth movement** (**b**). To allow a secure injection without potential harm to the animal by inadvertent movements or an otherwise required sedation, a custom-made acrylic box for short-time immobilization (20 s) was used. By means of a NiTi closed coil spring the upper left first and second rat molars (M1/M2) were moved in anterior direction. The contralateral jaw side (wire ligature) served as non-force control (split-mouth design).

The rats of the nicotine group (3) were subjected to a daily dose of 1.89 mg·kg^−1^ gross body weight of L(−)-nicotine (N3876, Sigma-Aldrich, St Louis, MO, USA; PubChemCID: 89594) throughout the respective experiment between 8:00 am and 12:00 am, dissolved in 0.9% isotone phosphate-buffered saline (PBS, pH=7.4). For this purpose the rats were immobilized short term (20 s) within a custom-made acrylic box and the nicotine solution injected into a skin fold pulled gently from an opening caudal of the rats’ neck area (Figure 2a). Administration was initiated 10 days before orthodontic treatment to allow a steady-state bioavailability^10^ simulating a long-term nicotine exposure. Within the first five days of administration, the nicotine dose applied was increased daily from 1/5 of the final dose in 1/5 increments to allow the animals to adapt.^10^ The rats of the remaining groups (1–2) were treated with PBS only (vehicle). To monitor animal welfare, all adverse events and gross body weight were registered on a daily basis (score sheet).

#### Orthodontic treatment

After a pre-exposure period of 10 days, we initiated orthodontic tooth movement in all rats except controls under short-term xylazine/ketamine sedation inserting a modified nickel-titanium closed coil tension spring (0.25 N, Sentalloy, GAC International, Graefelfing, Germany, 10-000-26) according to Kirschneck *et al.*^2, 10, 20^ This spring connected the base of the upper incisors (wire ligature, dental composite) to a shared cervical wire loop (Ø 0.01”) around the upper left first (M1) and second (M2) molars, resulting in anterior orthodontic tooth movement of both molars (Figure 2b). A constant force (0.125 N per tooth) could be maintained throughout the experiments, confirmed by an orthodontic calibrated spring balance (Correx, small model, Haag-Streit AG, Köniz, Switzerland) at insertion and end of treatment. At the contralateral jaw side we inserted an additional joint wire loop to correct for possible bias in tooth movement quantification (non-force-control, split-mouth design). To prevent mechanical damage to the coil spring, we cut the lower incisors at the papillary level on a weekly basis with a diamond-plated rotating disc and cleaned the spring at the same time (chow residues).

#### The act of killing

The rats were killed according to legal guidelines 42 (exp. A) and 14 days (exp. B/C) after the insertion of the coil spring, with an i.p. injection of pentobarbital sodium 1 h after injection of the last nicotine dose to ensure comparable serological results (200 mg·kg^−1^, Narcoren, Merial GmbH, Hallbergmoos, Germany).

### Experimental procedures

#### Experiment (A) Orthodontic tooth movement (CBCT)

To evaluate the extent of orthodontic tooth movement, we recorded radiological CBCT 3D images according to Kirschneck *et al.*^20^ (Veraviewepocs 3D R100/F40, Morita, Kyoto, Japan; 90 kV/5 mAs/9.4 s) both after the placement of the coil springs and after 14 and 28 days with the rats in xylazine/ketamine sedation.

**Figure 3:**
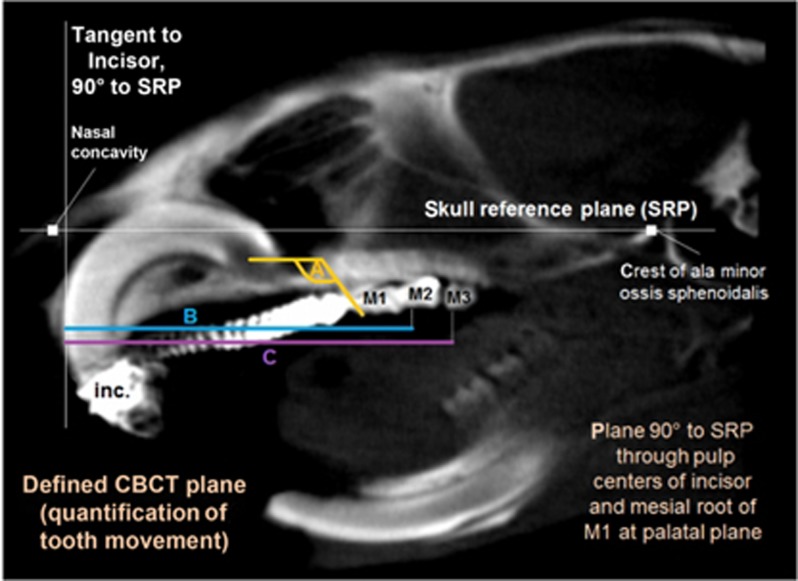
**CBCT.** Tooth movement was quantified within a defined two-dimensional plane of the rat skull in relation to a skull reference plane (SRP) and bias-corrected via the contralateral jaw side: (**a**) mesial angular tipping of first upper molar (M1), (**b**, **c**) mesial movement of second (**b**) and third (**c**) upper molars (M2/M3). inc., incisors. CBCT, cone-beam-computed tomography.

As described by Kirschneck *et al.,*^20^ tooth movement was quantified within a reproducible plane of the blinded CBCT data at the orthodontically treated left jaw side, defined with the software OneVolumeViewer (ver. 1.7.3, Morita) within the BONE1-window (WL 1000, WW 3000). A corresponding contralateral measurement at the non-force side was used for bias-correction by subtraction. The following types of tooth movement were recorded (Figure 3):

1. Mesial tipping of the first upper left molar (M1): decreasing angle between a static skull reference plane (SRP) and the connection line of the mesial cusp and root apex.
2. Mesialisation of the second (M2) and mesial drift of the third (M3) upper left molar: decline in distance between a tangent to the upper incisors and the respective mesial cusp apex (quantified parallel to the SRP).

Test–retest reliability was tested via two CBCT volumetric datasets taken and analyzed per animal at the start of orthodontic treatment. In addition, intra- and inter-rater reliability was checked for all measurements, which were repeated by the same and a different blinded researcher after a minimum of 2 weeks.

#### Experiment (B) Dental root resorption, osteoclast activity (histology) and IL-6 serology

After the injection of pentobarbital sodium, blood was collected from the left cardial ventricle, immediately processed to serum according to an established protocol and stored at −80 °C up to 1 week until serological testing. Subsequently, a perfusion-fixation was performed via the ascending aorta with 250 mL 4% paraformaldehyde in 0.1 mol·L^−1^ phosphate-buffered saline (PBS). We retrieved the left upper alveolar processes and subjected them to further immersion-fixation for 48 h.^2, 18, 22^ After a demineralization phase of 12 weeks in Tris-buffered EDTA solution (10%, pH=7.4) at room temperature, the alveolar processes were embedded in paraffin and sections of 2 μm were prepared in transversal direction. We used the slice with the maximum length of the distobuccal root of the second molar of each specimen for TRAP histochemistry^2, 22^ to evaluate osteoclast activity within the periodont (hematoxylin-eosin-counterstaining).^2, 18, 22^ All chemicals were ordered from Sigma-Aldrich.

**Figure 4:**
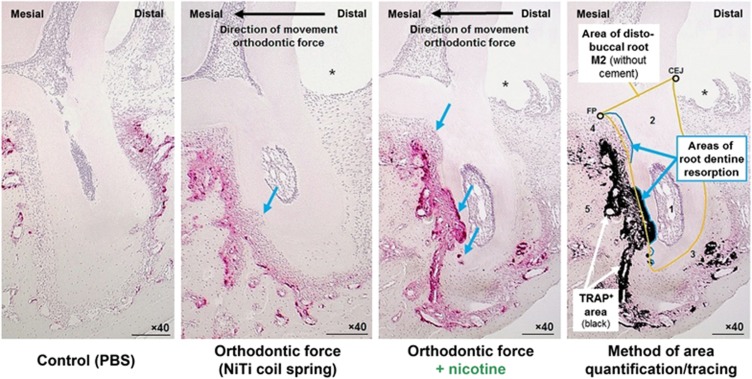
**Histological sagittal-oblique sections of the distobuccal root of the upper left second rat molar (M2) after 14 days of tooth movement (TRAP-staining, × 40, scale bars: 200 μm)**. ImageJ-traced TRAP+ area (red-violet) is shown in black. 1, Pulpa dentis (dental pulp); 2, Dentinum (dentine); 3, Substantia ossea dentis (Cementum, cement); 4, Desmodontium (periodontal ligament); 5, Os alveolare (alveolar bone); * former location of wire traction ligature; blue arrows=dentine root resorption areas; CEJ, cemento-enamel junction; FP, furcation point; PBS, phosphate-buffered saline; TRAP, tartrate-resistant acid phosphatase. *n*=7 (number of samples per experimental group).

After digitalization at × 40 magnification (Olympus BX45TF microscope with SC30 CMOS camera 3.3 MP, Olympus Deutschland GmbH, Hamburg, Germany), we calculated the relative TRAP-positive area within the periodontal ligament of the distobuccal root of M2 and relative dentine root resorption using the software ImageJ (ver. 1.47, Wayne Rasband, National Institutes of Health, USA):^2, 18^ We divided the absolute TRAP^+^ area and the total dentine resorption area (both in pixel) by the total distobuccal root area within the same slice (in pixel, dentine and pulp) (Figure 4). TRAP^+^ area tracing was performed automatically at a defined color threshold (brightness 0–255; hue 215–255; saturation 85–255), whereas dentine resorption and total root areas were traced by hand.^2, 18^ The crown-root demarcation line was defined by the cemento-enamel-junction (CEJ) and the furcation point (FP).^2, 18, 22^ Manual tracings were done twice with an interval of at least 2 weeks by the same and a different blinded researcher for evaluation of intra- and inter-rater reliability.

To assess systemic effects of the nicotine after 14 days of OTM, serum concentration of interleukin 6 (IL-6, ng·mL^−1^) was quantified by ELISA (HZ-EK0224, Cymax Rat IL-6 ELISA, Hölzel Diagnostika GmbH, Köln, Germany) in five randomly selected animals per experimental group. Reliability of enzyme linked immunosorbent assay (ELISA), performed according to the manufacturer’s instructions, was confirmed by non-antibody and non-template controls (NACs/NTCs).

#### Experiment (C) Relative gene expression (RT–qPCR) and cotinine serology

After collection of standardized cuboid samples of dental-periodontal tissue including the upper first and second molars without the clinical crown after 14 days of orthodontic treatment,^21^ they were immediately flash-frozen in liquid nitrogen, homogenized and total RNA was extracted with peqGOLD TriFast (PEQLAB Biotechnologie GmbH, Erlangen, Germany) as instructed by the manufacturer.

To evaluate purity and quantity, we performed photometry at 280, 260 and 230 nm after elution of the RNA pellet in 25 μL nuclease-free water with 1 OD_260nm_ corresponding to 40 μg·mL^−1^ total RNA.^21^ Whereas protein-free RNA can be assumed by an OD_260nm/280nm_ ratio of >1.8, phenol-/ethanol-free RNA is indicated by an OD_260nm/230nm_ ratio of >2.0.^21^

After transcribing the RNA in cDNA,^21^ we performed quantitative PCR (SYBR Green) and determination of C_q_ values of all reference and target genes (Supplementary Table S1) with a Mastercycler ep realplex-S Thermocycler and the software realplex (ver. 2.2, CalqPlex algorithm, automatic baseline, drift correction on; Eppendorf AG, Hamburg, Germany). Amplification was done in triplet for each tissue sample within the same qPCR plate (1 μg RNA equivalent per well).^21^ Thus bias by inter-run variations could be avoided.^21^ After transformation of the mean C_q_ values from each triplet to linear quantities (Q) in consideration of the qPCR efficiency (E) as Q=E^−(Cqmin-Cqsample)^, we normalized gene expression via the geometric linear quantity mean of the reference genes PPIB and YWHAZ.^21^

For assessment of qPCR efficiency (*E*) and validity (*R*^2^) a cDNA dilution series (standard curve) was created and values of 90%≤*E*≤100% and *R*^2^>0.98 accepted.^21^ To determine qPCR specifity of the in-silico specific primers a melting curve analysis (MCA, specific peak) and agarose gel electrophoresis (single band, correct molecular weight) was performed.^21^ Technical (intraassay) reliability was assumed if the highest C_q_ SD of the technical triplets did not exceed 0.553.^21^ Primer dimer formation was accepted as negligible, if no-cDNA-template controls (NTCs) showed *C*_q_ values >40 and no specific MCA peak was present.

To assess nicotine bioavailability after 14 days of OTM, serum cotinine concentration (μg·L^−1^) was quantified by GC-MS (gas chromatography-mass spectrometry) in five randomly chosen animals per experimental group. For this purpose, blood was retrieved and processed to serum as described before.

### Statistical methods

The software IBM SPSS Statistics 23 (IBM, Armonk, NY, USA) was used for all statistical analyses. For descriptive statistics means (M) and SD were calculated. *A priori* testing of all data with regard to normal distribution (Kolmogorov–Smirnov/Shapiro–Wilk test, visual assessment of histograms), variance homogeneity (Levene’s test, zpred *vs* zresid plots) and, if applicable, sphericity (Mauchly’s test) was performed. We used either mixed two-way (time/intergroup, CBCT data and body weight) analysis of variance (ANOVA) or independent two-sided one-way ANOVAs in conjunction with Tukey HSD *post hoc* tests. If significant intergroup variance heterogeneity was present, ANOVAs were corrected by Welch’s test and *post hoc* tests according to Games-Howell calculated. If data deviated significantly from normality, we performed non-parametric Kruskal–Wallis *H* and Mann–Whitney *U* follow-up tests. In case of a violation of sphericity, a Greenhouse-Geisser correction was used. A global MANOVA (Pillai’s trace *V*) was calculated for gene expression data.

*P*≤0.05 was defined as statistically significant and effect sizes (Pearson’s correlation coefficient *r*) were determined with *r*>0.5/0.3/0.1 corresponding to a large, medium or small mean difference or effect. Lin’s concordance correlation coefficient CCC was used to evaluate intra- and intergroup reliability. *ρ*_c_≥0.99 was defined as almost perfect agreement, 0.95≤*ρ*_c_<0.99 as substantial, 0.90≤*ρ*_c_<0.95 as moderate and *ρ*_c_<0.90 as poor level of agreement.

## Results

### Animal welfare, adverse events and numbers analyzed

All 63 rats were available for analysis of the target variables, although one animal of experiment A (nicotine group) died after the experimental phase on day 35. The rats remained in good health with gross body weight steadily increasing (except directly after the orthodontic intervention) from day 10 (onset of nicotine administration, M=246 g; SD=14 g) over day 0 (start of OTM, M=260 g; SD=15 g), day 14 (M=259 g; SD=21 g) and day 28 (M=272 g; SD=24 g) of tooth movement: F=46.655; df=2.049; *P*≤0.001. Gross body weight of the nicotine-treated rats, however, was significantly reduced compared to other groups on day 0, 14 and 28: F=19.342; df=2; *P*≤0.001. The desired force level of 0.25 N was corroborated at beginning and end of treatment. We observed no adverse events, except mild short-term agitation of animals directly after nicotine administration. No changes to the experimental design were made during the course of the study.

### Serum concentration of interleukin-6 and cotinine

After 25 days of daily nicotine administration (day 14 of tooth movement), blood serum concentration of interleukin 6 was significantly higher in nicotine-treated animals than in PBS-treated animals (Table 1, Supplementary Figure S1) by a factor of at least x2.6 (mean). Whereas the PBS controls had cotinine serum levels below the detection threshold of 5 μg·L^−1^, a pronounced mean cotinine serum concentration of 458.8 μg·L^−1^ was found for the experimental group exposed to nicotine (Table 1, Supplementary Figure S1).

### Orthodontic tooth movement velocity and mesial drift

Orthodontic therapy caused significant anterior tooth movement of the upper molars M1/M2 as well as mesial drift of M3 (Table 1,Supplementary Figure S3). Over the course of time from 14 to 28 days, the traversed distance increased significantly, both for M1 (F=59.639; df=1; *P*<0.001; *r*=0.77; Table 1), M2 (F=219.362; df=1; *P*<0.001; *r*=0.96; Table 1) and M3 (F=40.210; df=1; *P*<0.001; *r*=0.83; Table 1). Under the influence of nicotine, a significant acceleration of mean tooth movement velocity (corrected via the contralateral non-force jaw side) was observed (Table 1, Supplementary Figure S3), both for mesial tipping of the first upper left molar (M1, day 14+71%, day 28+55% F=91.183; df=2; *P*<0.001; *r*=0.95) and for mesial movement of the second upper left molar (M2, day 14+51%, day 28+52% F=125.398; df=2; *P*<0.001; *r*=0.97). We also found a significant acceleration of mesial drift of the third upper left molar (M3) into the gap evolving between the second and third molar in nicotine-treated animals by approximately +82% (mean, day 14) and +75% (mean, day 28): F=40.659; df=2; *P*<0.001; *r*=0.90. Substantial concordance was corroborated for all radiological distances measured regarding intra- and inter-rater as well as test-retest reliability (*ρ*_c_>0.95).

### Dental-periodontal gene expression of inflammatory and osteoclast markers

Compared to orthodontic therapy by itself, the combination of orthodontic force and nicotine administration caused a significant increase in mean relative normalized gene expression within the dental–periodontal tissue of the moved first and second upper molars on day 14 after the orthodontic intervention (Table 1, Supplementary Figure S2; V=1.426; F=6.953; *P*<0.001) for the investigated osteoclast markers cathepsin K (CTSK) (13.2x) and CLCN7 (5.2x) as well as the inflammatory cytokines IL-1β (4.0x), IL-6 (3.2x) and IL-8/CXCL1 (2.1x). Technical reliability, validity, efficiency and specifity of qPCR amplification each conformed to the prespecified range and limits deemed acceptable (Supplementary Table S2). Satisfactory purity of the extracted RNA was achieved as indicated by OD_260/280_ ratio of 1.93 (SD 0.06) and OD_260/230_ ratio of 2.02 (SD 0.07).

### Periodontal osteoclast activity

On day 14 of orthodontic therapy a significantly more pronounced relative TRAP-positive area (× 1.6, mean), particularly within the periodontal compression areas (distobuccal root of M2), was observed within the animals, which had received a daily dose of nicotine in addition to orthodontic force application (Figure 4, Table 1). Whereas a concentration of TRAP^+^ activity and osteoclasts was found at the alveolar bone surface in direction of tooth movement within the animals treated only orthodontically (PBS), a more even distribution was observed within the animals treated with nicotine, with osteoclasts invading the dentine root surface in extended resorption lacunas.

### Extent of root resorptions (dentine) at the distobuccal root of M2

OIIRR was found histometrically in all rats with experimental tooth movement after 14 days of orthodontic therapy at the distobuccal root of second upper molar M2 (Figure 4, Table 1). In animals treated with nicotine, the extent of these OIIRR was significantly increased by a factor of × 3.7 (mean). Intra- and inter-rater reliability was substantial for all manual tracings performed (*ρ*_c_>0.95).

## Discussion

With our *in vivo* study we wanted to investigate possible undesired effects of chronic nicotine exposure at a dosage corresponding to that of an average European smoker. Our results indicate that nicotine significantly accelerated orthodontic tooth movement and increased associated undesired OIIRR as well as exponentiated the underlying osteoclast activity and inflammation within the periodontal ligament.

Nicotine is generally absorbed into the human body by the inhalation of cigarette smoke within a few seconds, where its systemic stimulatory and psychoactive effect unfolds by binding to cell membrane-based nicotinic acetylcholine receptors (nAChR) of the nervous system.^13, 23^ Chemo-analytical methods, however, have shown that nicotine is also enriched in fibroblasts of the periodontal apparatus^24^ and the root surfaces of teeth^25^ during tobacco consumption, suggesting possible local effects in the periodontal tissues.

Several *in vivo* and *in vitro* studies have found that nicotine can have a proinflammatory effect on periodontal tissues and influence bone metabolism. Nicotine has been shown to dose-dependently increase the expression of cyclooxygenase 2 (COX-2) in human gingival and periodontal ligament fibroblasts.^10, 26^ Furthermore, several studies have indicated that prostaglandin E2 enhances the expression of proinflammatory cytokines by fibroblasts, in particular of IL-1β, IL-6 and IL-8.^27, 28^ A nicotine-induced increase in the production of prostaglandin E2 could thus provide an explanation for the significant nicotine-induced increase in interleukin expression observed.

The major mechanism for osteoclast activation and differentiation is the interaction of RANKL with the RANK-receptor of osteoclast precursor cells.^3^ IL-1β and IL-6 have been reported to induce RANKL synthesis by periodontal ligament fibroblasts both via an paracrine and autocrine mechanism.^3, 4^ A consecutively increased RANKL expression within the dental-periodontal tissue ^10, 11, 29^ could explain the increased number of osteoclasts and TRAP^+^ activity observed in compression zones of the periodontal ligament in our histological slides of the alveolar process. It would also explain the observed reduction in gene expression of CTSK, a cysteinproteinase released by osteoclasts, which degrades the extracellular matrix of bone, and of CLCN7, a chloride ion channel within the osteoclast cell membrane. This mechanism of action is supported by several previous studies indicating a stimulatory effect on bone catabolism^11, 30, 31^ and inhibitory effect on bone anabolism.^11, 32, 33^ Furthermore, periodontal immigration of B- and T-lymphocytes, which are primary sources of RANKL^34^ during periodontitis, may have been significantly reduced by the observed decline of IL-8 expression (chemokine).^35^

Orthodontic tooth movement, on the other hand, is also enabled by a similar, but controlled (pseudo)inflammatory process within the periodontal ligament and bone.^3^ Proinflammatory cytokines and signalling molecules are released by mechanical deformation of the involved cells (periodontal ligament fibroblasts, osteocytes and others) and tissue after force application (mechanotransduction).^3^ As a consequence, a reconstruction processes is instigated within the periodontal tissue and adjacent bone as well as a change in blood flow and vascularization.^3, 36, 37^ This leads to an increase in osteoclastogenesis and bone resorption in compression zones of the periodontal ligament, whereas the recruitment of osteoblasts with corresponding osteogenesis is increased in tensile zones, thus enabling stable tooth movement overall.^3^

The nicotine-induced increase in osteoclast activity and osteoclastogenesis explains the observed nicotine-induced increase of OIIRR as well as acceleration of orthodontic tooth movement within 14 and 28 days. We suggest based on our observations that nicotine exposure during orthodontic tooth movement synergistically increased the release of proinflammatory cytokines and thus RANKL-mediated differentiation of osteoclasts within the compression areas of the periodontal ligament, resulting in increased resorption of both alveolar bone in direction of movement and formation of OIIRR. In addition, the previously observed progressing loss of periodontal bone^10^ and thus higher relative forces exerted on the periodontal ligament as well as reduced bone density (bone volume/trabecular thickness)^38^ over time could also have contributed to the observed tooth movement acceleration. This acceleration would be a desirable effect in orthodontic treatment, since it could reduce treatment time and associated risks such as white-spot lesions (initial caries) and gingivitis, which increase with treatment duration.^39^ Research on suitable pharmacological substances and their safe delivery and usage to this end is currently intensively pursued.^40, 41^ Although nicotine could be administered systemically in a controlled fashion via a nicotine patch or administered locally by injection into the periodontal ligament,^41^ the severe detrimental side effects observed and to be expected (root resorptions, periodontal bone loss) as well as the clinically limited acceleration achieved (about 50%) most likely exclude nicotine as suitable drug for possible adjuvant therapeutic use in orthodontics.

A common, but unwelcome side effect, which may occur during orthodontic tooth movement, are orthodontically induced inflammatory OIIRR.^5, 6^ We found a distinct and significant increase in both extent and number of resorption areas of the dental root in compression zones of the periodontal ligament. This was probably due to the increased osteoclast activity, which are virtually identical to the so-called “odontoclasts” responsible for dental root resorption, both in function and ultrastructure.^42^ Furthermore, the processes of root resorption and bone remodelling involve the same receptor ligand system known as RANK/RANKL.^5, 6^ Several factors are considered to influence the extent of root resorptions: the force level used, the duration of treatment as well as the velocity and degree of orthodontic tooth movement achieved.^5, 6^ Since the latter was significantly increased in our study, this may have contributed to the degree of tooth movement observed. In young rats, however, this effects was found to be rather small, especially within the limited treatment time of our study.^43^

Little is yet known about the interaction of nicotine and orthodontic tooth movement. In a previous study,^10^ we found that orthodontic force application and tooth movement in a rat model significantly increased nicotine-associated periodontal bone loss and that nicotine dose-dependently enhanced the release of proinflammatory cytokines and prostaglandin E2 by periodontal ligament fibroblasts stressed by orthodontic forces *in vitro*. Much information is available on the effects of nicotine on the periodontal ligament itself. Various studies report an association between chronic tobacco or nicotine consumption and the occurrence of periodontitis as well as a loss of structures of the periodontal apparatus,^11, 12, 15^ but the exact mechanism, how nicotine induces an upregulation of inflammatory processes and consecutively osteoclastogenesis and bone resorption is still elusive.

One proposed mechanism of action might be the interaction of nicotine with the nicotinic acetylcholine receptor (nAChR) of periodontal cells, which is functionally expressed as α7 subtype in fibroblasts and tissue of the periodontal ligament and seems to induce the expression of proinflammatory cytokines via the NFκB pathway.^14, 19, 44^ These studies also showed that antagonization of this receptor by mecamylamine or α-bungarotoxin led to a reduction or even neutralization of the periodontally harmful influence of nicotine.^14, 19^

Alternatively, the heightened inflammatory response within the periodontal ligament could also be triggered by the activation of nAChR receptors of the nervous system and subsequent stress reaction of the central nervous system (release of adrenaline). This is supported by results from Takada *et al.*^45^ and El Attar *et al.*,^46^ who found that stress can cause loss of periodontal attachment and demonstrated that adrenalin elevated gingival prostaglandin E2 levels in patients suffering from chronic periodontitis. However, studies indicate that nicotine appears to evoke an adaptation of the hypothalamic pituitary system in the long term, resulting in normal glucocorticoid concentrations in the blood.^47^ Thus a systemic stress reaction by itself is probably not the main perpetrator of the periodontal inflammatory and decomposition processes. Benatti *et al.*, however, found that stress may at least enhance nicotine-induced detrimental effects on periodontal tissue.^48^

A third possible mechanism of action explaining the nicotine-associated exponentiating effect on orthodontic tooth movement and dental root resorption involves a possible local vasoconstriction of blood vessels and/or reduced angiogenesis described in the literature,^49, 50^ which could result in periodontal hypoxia. Since orthodontic forces can also lead to circulatory disturbances in the periodontal ligament due to compression of the blood vessels ^36^ and are assumed to cause hypoxic states,^37^ orthodontically induced hypoxic effects may have been exponentiated by nicotine-induced hypoxia. This hypothesis was supported by observations from Kim *et al.*,^51^ who found that nicotine stimulated the synthesis of prostaglandin E2 and MMPs via enhanced expression of the hypoxia-inducible factor (HIF) 1α in periodontal ligament cells. It is also known that cyclooxygenase 2 (COX-2), whose expression by force-stimulated periodontal ligament fibroblasts was significantly enhanced in presence of nicotine,^10^ is a target gene of HIF-1α,^52^ which is the central mediating factor for the adaptation of tissues to hypoxic states.^53^ In addition, synergistically enforced hypoxic conditions within the periodontal ligament may favor the growth of the mostly anaerobic periodontal pathogens, responsible for bacterially induced periodontitis.^13^

The significantly reduced body weight of the nicotine-treated animals at all times was observed before^54^ and indicates in conjunction with the cotinine serum levels observed that the intended nicotine bioavailability was achieved during the experiment. Overall welfare of the animals was not adversely affected by nicotine, as body weight increased in both experimental groups. The experimental setup and model used is an established model for orthodontic tooth movement.^2, 18, 20^ For the rat, a subcutaneous administration of nicotine is described in the literature as an established method for simulating inhaled nicotine intake during smoking,^14, 19, 23^ which leads blood plasma levels corresponding to those found in chronic smokers, as evidenced by the serum cotinine levels observed.^55^

We calculated the administered nicotine dosage to correspond to the nicotine exposure of an average smoker in Europe (14.2 cigarettes per day) according to the European Commission.^56^ Starting from a mean nicotine content per cigarette of 1.533 mg (mean of 54 cigarette brands investigated by Moore *et al.*^57^) and an average body weight of 70.8 kg in Europe,^58^ the human equivalence dose (HED) was calculated as 0.307 mg per day per kg body weight, which corresponds to a rat equivalent dose (RED) of 1.89 mg per day per kg body weight considering an adjustment factor based on human-rat weight and body surface differences.^59^ Furthermore 1–4 mg per day per kg nicotine have been used in subcutaneous application in rat studies on nicotine before,^10, 15^ since stable plasma nicotine levels were achieved corresponding to those of habitual smokers.^23^ The successive increase in nicotine dose at the beginning was chosen to avoid an acute adrenergic effect on the cardiovascular system.

A limitation of our study is the fact that only one dosage of nicotine was tested. Further investigations are needed to determine dose–effect relationships to assess orthodontic and dental risks for occasional smokers, during smoking cessation therapy (nicotine patch) or for nonsmokers by environmental tobacco smoke.^17^ In addition, the exact pharmacodynamic mechanism of action, how nicotine-related effects are produced within the context of orthodontic treatment, requires additional research. Furthermore, it would be of clinical interest to know the minimally required nicotine abstinence period before the start of orthodontic therapy, which allows safe orthodontic tooth movement, which should be investigated in future studies.

Caution is generally required when attempting to translate results from animal experiments to humans. Our results on nicotine and tooth movement, however, should allow sufficient translatability, since rats are the “primary preclinical model for human nicotine exposure”^23^ and the standard animal model for studying the effects of orthodontic tooth movement.^60^ Although some differences exist regarding the velocity of nicotine metabolism (faster in rats and disparity in primary cytochrome P450 enzyme), neurotransmitter mechanisms and nAChRs are quite similar.^23^ However, since nicotine is only one of over 4 000 components present in tobacco smoke,^10^ the results obtained in this study are most likely translatable to pure nicotine consumption, such as via a nicotine patch used for smoking cessation, but not necessarily generalizable to the smoking patient in general. Recent findings in an animal study by Nagaie *et al.*^61^ indicate that tobacco smoke as a comprehensive mixture of numerous components with tar being the most prevalent, may actually retard orthodontic tooth movement. These results would indicate that other components in tobacco smoke apart from nicotine actually exert an inhibiting effect on tooth movement and osteoclast activity, most likely due to their cytotoxic effects, which were shown by the authors to be 100-fold higher than purely nicotine-associated cytotoxic effects.^61^ In smoking patients these other components may possibly overcompensate the accelerating effect of nicotine observed in our study. However, our study was not able to clarify if the distinctly lower nicotine doses, which were used in the study by Nagaie *et al.*^61^ (about 20.1 μg nicotine per animal per day), actually elicit a clinically relevant acceleratory effect on orthodontic tooth movement via the mechanisms discussed previously. Thus clinical effects of nicotine and tobacco smoke on orthodontic tooth movement may be dose dependent, possibly ranging from no effect by low doses, an accelerating effect by higher doses to even inhibiting effects by cytotoxic doses—a hypothesis, which will need to be further investigated in future studies.

## Conclusions

During orthodontic tooth movement in the presence of nicotine at a dosage corresponding to that of an average European smoker, an exponentiation of OIIRR and accelerated orthodontic tooth movement are to be expected in addition to the previously observed increase in periodontal bone loss. Although the achieved acceleration of tooth movement would be desirable for treatment purposes to reduce total treatment time and associated orthodontic treatment risks, the observed severe side effects indicate the need to properly inform orthodontic patients about the risks and the necessity of nicotine abstinence during orthodontic treatment, which should only be started after complete cessation of nicotine consumption. Since tobacco smoke, however, consists of many more pharmacologically active components than nicotine itself, our effects observed in an animal model at a particular nicotine dosage should be clinically translatable to direct nicotine intake, for example, via a nicotine patch, but need not necessarily be generalizable to smokers or tobacco-consuming patients in general or to other dosages of nicotine intake.

## Figures and Tables

**Table 1:** Descriptive data and statistics of all outcome parameters evaluated

		Descriptive statistics				Global test					*Post hoc* test Δ force (F)/F+nicotine	
Parameters Evaluated	Experimental conditions (*n*=7 per group)		M	SD	min.	max.	F/H/U	df_1-2_ (z)	*P*-value	*r*	*P*-value	95% CI of ΔM
*Serology (after 14 days of OTM, nicotine bioavailability (cotinine) and systemic inflammation (IL-6))*												
IL-6—blood serum concentration in ng·mL^−1^ (*n*=4/grp.)	Control (PBS)		0.9	0.7	0.1	1.7	17.407^F^	2	***0.001	0.89	**0.009^**T**^	1.6–9.9
	Orthodontic force		3.7	0.8	2.5	4.3						
	Force+nicotine		9.5	3.5	6.3	14.3						
Cotinine—blood serum concentration in μg·L^−1^ (*n*=5/grp.)	Control (PBS)		5.0	0.0	5.0	5.0	25.000^U^	2.785^z^	**0.008	0.88	**0.008^**U**^	283.7–623.9
	Force+nicotine		458.8	137.0	258.0	569.0						

*CBCT (radiology, experiment A, OTM within 14/28 days, velocity of orthodontic tooth movement)*												
First upper left molar M1—orthodontic tooth movement within 14/28 days (tipping, in °)	Control (PBS)	14 days	−0.3	0.9	−1.6	0.8	91.183^F^ (group) 59.639^F^ (time)	2 (group) 1 (time)	***<0.001 (group) ***<0.001 (time)	0.91 (group) 0.88 (time)	***<0.001^**T**^	1.4–4.5
		28 days	0.0	1.5	−1.8	2.0						
	Orthod. force	14 days	3.6	1.6	1.7	5.3						
		28 days	6.3	1.5	4.1	7.8						
	Force+nicotine	14 days	6.1	1.2	4.5	7.9						
		28 days	9.7	1.1	8.5	11.4						
Second upper left molar M2—orthodontic tooth movement within 14/28 days (in mm)	Control (PBS)	14 days	0.0	0.0	0.0	0.1	125.398^F^ (group) 219.362^F^ (time)	2 (group) 1 (time)	***<0.001 (group) ***<0.001 (time)	0.94 (group) 0.96 (time)	**0.004^**GH**^	0.1–0.4
		28 days	0.0	0.1	−0.1	0.1						
	Orthod. force	14 days	0.4	0.1	0.3	0.5						
		28 days	0.7	0.1	0.6	0.8						
	Force+nicotine	14 days	0.6	0.1	0.5	0.7						
		28 days	1.0	0.2	0.8	1.3						
Third upper left molar M3—mesial drift within 14/28 days (in mm)	Control (PBS)	14 days	0.0	0.0	0.0	0.1	40.659^F^ (group) 40.210^F^ (time)	2 (group) 1 (time)	***<0.001 (group) ***<0.001 (time)	0.83 (group) 0.83 (time)	**0.002^**T**^	0.1–0.3
		28 days	0.0	0.1	−0.2	0.2						
	Orthod. force	14 days	0.1	0.1	0.0	0.2						
		28 days	0.3	0.1	0.2	0.5						
	Force+nicotine	14 days	0.2	0.1	0.2	0.3						
		28 days	0.5	0.1	0.4	0.6						

*RT–qPCR (after 14 days of OTM, experiment B, inflammation and osteoclast activity within the dental-periodontal tissue)*												
IL-1β—normalized relative gene expression (geNorm)	Control (PBS)		0.04	0.03	0.02	0.10	42.52^F,W^	2–7.301	***<0.001	0.92	**0.002^**GH**^	0.6–1.9
	Orthodontic force		0.42	0.22	0.16	0.75						
	Force+nicotine		1.65	0.57	0.71	2.26						
IL-6—normalized relative gene expression (geNorm)	Control (PBS)		0.01	0.01	0.00	0.02	32.93^F,W^	2–7.301	***<0.001	0.90	***<0.001^**GH**^	0.6–1.5
	Orthodontic force		0.47	0.17	0.29	0.74						
	Force+nicotine		1.53	0.39	1.03	2.05						

IL-8-equivalent (CXCL1)—normalized rel. gene expression (geNorm)	Control (PBS)		0.09	0.05	0.03	0.17	78.85^F,W^	2–7.379	***<0.001	0.87	**0.002^**GH**^	0.4–1.6
	Orthodontic force		0.89	0.27	0.62	1.23						
	Force+nicotine		1.90	0.49	1.18	2.64						
CTSK—normalized relative gene expression (geNorm)	Control (PBS)		0.04	0.01	0.04	0.05	33.103^F,W^	2–7.421	***<0.001	0.90	***0.001^**GH**^	2.1–5.6
	Orthodontic force		0.31	0.10	0.19	0.49						
	Force+nicotine		4.15	1.53	2.35	6.44						
CLCN7—normalized relative gene expression (geNorm)	Control (PBS)		0.03	0.01	0.02	0.04	67.400^F,W^	2–7.603	***<0.001	0.93	**0.007^**GH**^	0.5–2.4
	Orthodontic force		0.34	0.06	0.27	0.41						
	Force+nicotine		1.79	0.79	0.92	3.00						

*Histomorphometry and Histochemistry (M2 distobuccal root, after 14 days of OTM, experiment C, OIIRR and osteoclast activity)*												
TRAP^+^ area in % in PDL per mm^2^ root area (TRAP density)	Control (PBS)		3.8	2.1	1.0	6.9	33.692^F,W^	2–11.0	***<0.001	0.90	**0.002^**GH**^	4.7–17.7
	Orthodontic force		10.1	2.9	5.1	13.9						
	Force+nicotine		21.3	5.4	14.6	30.1						
Relative dentine root resorption in %	Control (PBS)		0.0	0.0	0.0	0.0	18.491^H^	2	***<0.001	0.95	***0.001^**U**^	4.4–9.9
	Orthodontic force		2.6	0.5	2.0	3.4						
	Force+nicotine		9.7	2.3	6.9	13.5						

CBCT, cone-beam computed tomography; CI, confidence interval; df, degrees of freedom; F, test statistic of one-way/mixed (CBCT) ANOVA; GH, Games–Howell *post hoc* test; H, test statistic of two-sided Kruskal–Wallis *H-*test; IL, interleukin; M, arithmetic mean; max., maximum; min., minimum; *n*, number of animals; OTM, orthodontic tooth movement; OIIRR, orthodontically induced inflammatory root resorption; *P*, significance value; *r*, effect size (Pearson’s correlation coefficient); PDL, periodontal ligament; PBS, phosphate-buffered saline; RT–qPCR, reverse transcription quantitative real-time; PCR, polymerase chain reaction; SD, standard deviation; T, Tukey’s Highly Significant Difference *post hoc* test; TRAP, tartrate-resistant acid phosphatase; U, test statistic of two-sided Mann–Whitney *U*-test; W, Welch test corrected ANOVA; z, standardized test statistic; ΔM, difference of arithmetic means of group force (2) and group force+nicotine (3).

Gene expression was normalized via the reference genes PPIB/YWHAZ (combination). Bold values are indicate *P* values, which are statistically significant (*P*≤0.05). */**/***P≤0.05/0.01/0.001.
