# Supplementary material for: Regular nicotine intake increased tooth movement velocity, osteoclastogenesis and orthodontically induced dental root resorptions in a rat model
Source: Int J Oral Sci. 2017 Sep 29;9(3):174–84. doi: 10.1038/ijos.2017.34 (PMC5709548; doi:10.1038/ijos.2017.34)
Supplement: Supplementary Table S1 [file ijos201734x1.pdf]

Table S1 RT-qPCR information on genes, primers and amplicons used for relative gene expression analyses.

**Gene symbol**	**Gene name**	**Accession Number**[1] (NCBI GeneBank)	**Chromosomal location** (length)	**5´-forward primer-3´** (length / T_m / %GC)	**5´-reverse primer-3´** (length / T_m / %GC)	**Primer Location**[2]	**Intron-spanning** (length)	**In silico qPCR specifity**[3]	**Splice variants targeted**[2] (total number)	**Amplicon** (length / %GC / T_m)	**Amplicon position (cDNA)** (bp start-stop)
**PPIB**	peptidylprolyl isomerase B	NM_022536.1	8q24 (840bp)	ACGTGGTTTTCGGCAAAGT (19bp / 60°C / 47%)	CTTGGTGTTCTCCACCTTCC (20bp / 59°C / 55%)	exon 4/5	yes (664bp)	yes	no (1)	62bp / 52% / 81.0°C	495-556
**YWHAZ**	tyrosine 3-monooxygenase /tryptophan 5-monooxygenase activation protein, zeta	NM_013011.3	7q22 (1815bp)	CTACCGCTACTTGGCTGAGG (20bp / 60°C / 60%)	TGTGACTGGTCCACAATTCC (20bp / 59°C / 50%)	exon 3/4	yes (609bp)	yes	yes (2)	63bp / 51% / 80.8°C	436-479
**IL1B**	interleukin 1 beta	NM_031512.2	3q36 (1339bp)	TGTGATGAAAGACGGCACAC (20bp / 60°C / 50%)	CTTCTTCTTTGGGTATTGTTTGG (23bp / 59°C / 39%)	exon 6/7	yes (718bp)	yes	yes (2)	70bp / 51% / 81.1°C	617-664
**IL6**	interleukin 6	NM_012589.1	4q11 (1046bp)	CCTGGAGTTTGTGAAGAACAACT (23bp / 59°C / 43%)	GGAAGTTGGGGTAGGAAGGA (20bp / 60°C / 55%)	exon 4/5	yes (142bp)	yes	no (1)	142bp / 37% / 79.6°C	433-555
**CXCL1 (IL8-equiv.)**	chemokine (C-X-C motif) ligand 1	NM_030845.1	14p22 (929bp)	CATTAATATTTAACGATGTGGATGCGTTTCA (31bp / 64°C / 32%)	GCCTACCATCTTTAAACTGCACAAT (25bp / 63°C / 40%)	exon 4	no	yes	no (1)	76bp / 38% / 78.7°C	493-568
**CLCN7**	chloride channel, voltage-sensitive 7	NM_031568.1	10q12 (2750bp)	TGTCCTACCTCACAGGAGCA (20bp / 59°C / 55%)	CTCCCATCAGGGCGTATTTA (20bp / 59°C / 50%)	exon 17/18	yes (1216bp)	yes	no (1)	60bp / 58% / 81.4°C	1657-1716
**CTSK**	cathepsin K	NM_031560.2	2q34 (1446bp)	CGACTATCGAAAGAAAGGCTATG (23bp / 59°C / 43%)	AAAGCCCAACAGGAACCAC (19bp / 59°C / 53%)	exon 4/5	yes (1944bp)	yes	no (1)	69bp / 48% / 80.4°C	430-498

PPIB/YWHAZ were used as reference genes for normalization. All primers (except CXCL1) were constructed with Roche®ProbeFinder 2.5 (Primer3/Version 1.1.4). Unmodified intron-spanning primers were constructed whenever possible, synthesized by EurofinsMWGOperonLLC (Huntsville/AL/USA) and purified by HighPurity-Salt-Free-Purification HPSF®.

T_m=melting temperature of primer/specific qPCR product (amplicon); %GC = guanine/cytosine content; bp = base pairs.

[1]Primer design based on this sequence. The database source was the NCBI Nucleotide database (http://www.ncbi.nlm.nih.gov/nuccore).

[2]determined with PrimerCheck (http://projects.insilico.us/SpliceCenter/PrimerCheck; SpliceCenter).

[3]determined *in silico* by Roche® ProbeFinder 2.5 (http://qpcr.probefinder.com/organism.jsp) or the UCSC In-Silico-PCR database (http://genome.ucsc.edu/cgi-bin/hgPcr).
