# Supplementary material for: Regular nicotine intake increased tooth movement velocity, osteoclastogenesis and orthodontically induced dental root resorptions in a rat model
Source: Int J Oral Sci. 2017 Sep 29;9(3):174–84. doi: 10.1038/ijos.2017.34 (PMC5709548; doi:10.1038/ijos.2017.34)
Supplement: Supplementary Table S2 [file ijos201734x2.pdf]

Table S2. Data on RT-qPCR validity, efficiency and technical reliability.

**Gene symbol**	**Slope**	**Efficiency E (%)**	**95% CI of Efficiency E**	**Coefficient of determination R^2** (adjusted)	**Intraassay reliability** SD of triplet C_q means* (mean, min./max.)
**PPIB**	-3.37	98.0	93.0 - 103.5	0.994	0.06, 0.00/0.26
**YWHAZ**	-3.28	101.9	98.6 - 105.4	0.998	0.07, 0.02/0.23
**IL1B**	-3.48	93.9	90.4 - 97.7	0.997	0.16, 0.01/**0.48**
**IL6**	-3.16	107.1	105.1 - 109.5	0.987	0.33, 0.08/**0.48**
**CXCL1** (IL8 equiv.)	-3.17	106.6	104.2 - 109.6	0.998	0.16, 0.03/0.46
**CLCN7**	-3.50	93.2	90.9 - 95.6	0.999	0.16, 0.04/0.38
**CTSK**	-3.29	101.3	98.0 - 105.1	0.997	0.05, 0.00/0.17

Data based on a standard curve from an 8x $\log_{10}$ cDNA dilution series of each gene/primer pair. For each gene and biological sample, gene expression was quantified in triplets within the same qPCR plate. * three technical replicates (triplet), across all tissue samples/biological replicates (n = 21). CI = confidence interval; SD = standard deviation; min. = minimum; max. = maximum. Coefficient of determination R^2 as measure for the relationship C_q value – RNA-concentration was calculated by linear regression. qPCR efficiencies (E) were obtained by the equation $E=[10^{(-1/slope)}-1]\times 100$ from the standard curve and are given as percentage.
