# Supplementary material for: Regular nicotine intake increased tooth movement velocity, osteoclastogenesis and orthodontically induced dental root resorptions in a rat model
Source: Int J Oral Sci. 2017 Sep 29;9(3):174–84. doi: 10.1038/ijos.2017.34 (PMC5709548; doi:10.1038/ijos.2017.34)
Supplement: Supplementary Figure S1 [file ijos201734x3.pdf]

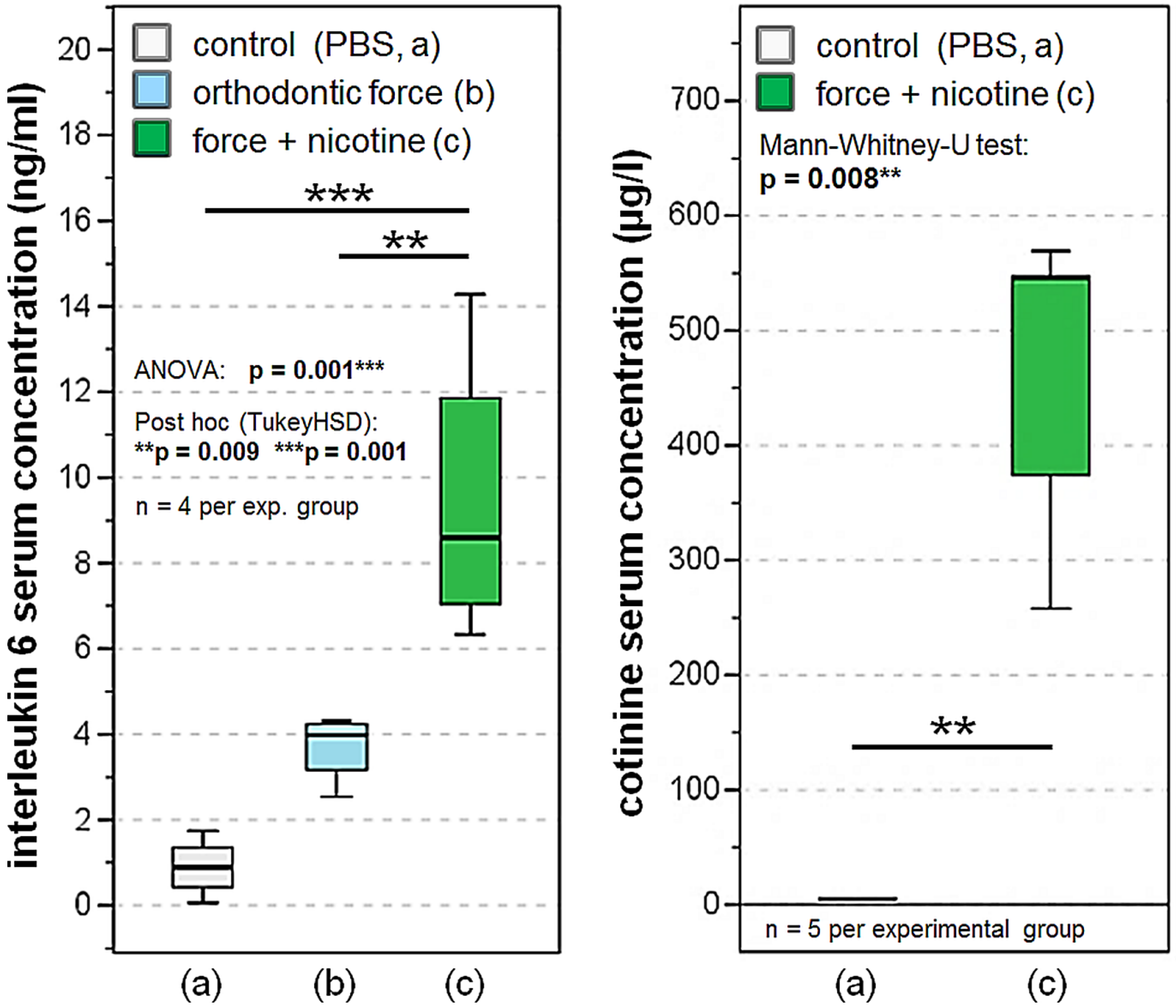

Figure S1 Interleukin 6 and cotinine (nicotine metabolite) concentration within the blood serum after 14 days of orthodontic therapy and 25 days of nicotine administration. n=4/5 (number of samples per experimental group). Boxplots show median and interquartile ranges, and whiskers denote the data range.
