# Supplementary material for: Regular nicotine intake increased tooth movement velocity, osteoclastogenesis and orthodontically induced dental root resorptions in a rat model
Source: Int J Oral Sci. 2017 Sep 29;9(3):174–84. doi: 10.1038/ijos.2017.34 (PMC5709548; doi:10.1038/ijos.2017.34)
Supplement: Supplementary Figure S2 [file ijos201734x4.pdf]

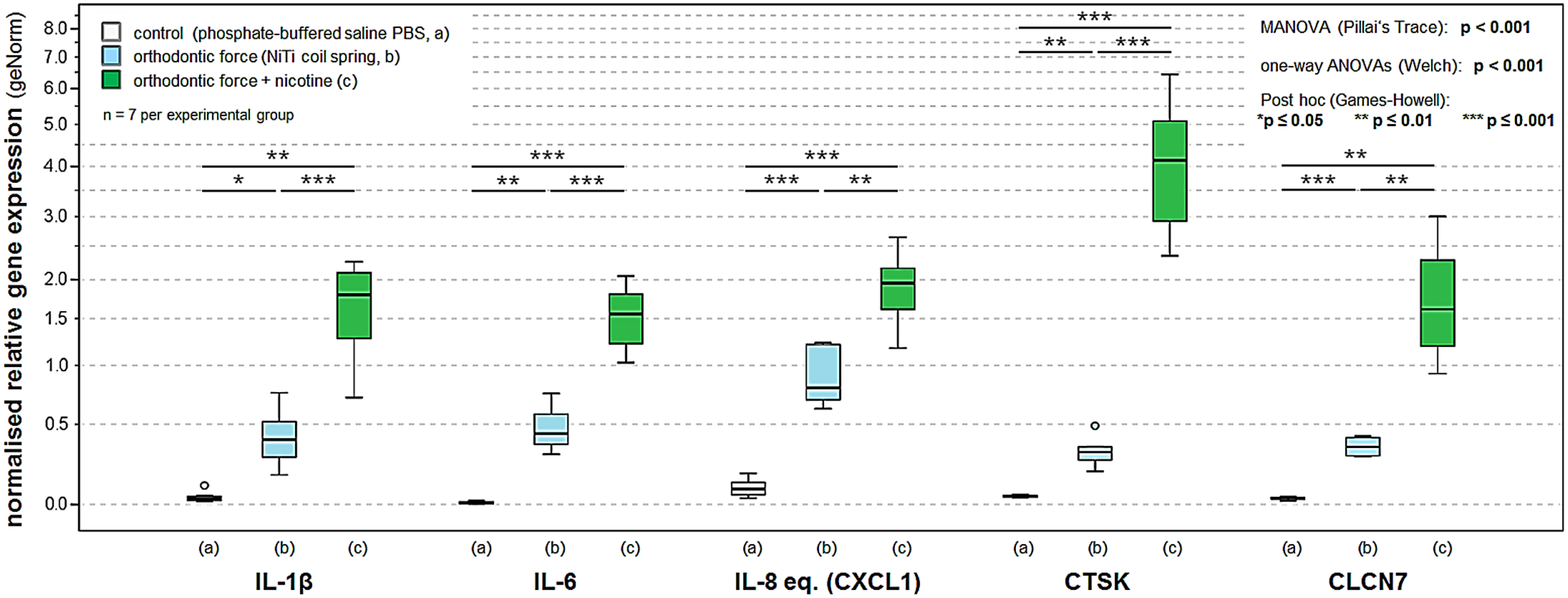

Figure S2 Relative normalized gene expression (three inflammatory and two osteoclast markers) after 14 days of orthodontic therapy in the dental-periodontal tissue of the first and second upper left rat molars (M1/M2). Abbreviations see Table S1. n=7 (number of samples per experimental group). Boxplots show median and interquartile ranges, and whiskers denote the data range.
